# Pharmacokinetics of Exenatide in nonhuman primates following its administration in the form of sustained-release PT320 and Bydureon

**DOI:** 10.1038/s41598-019-53356-2

**Published:** 2019-11-20

**Authors:** Yazhou Li, Kelli L. Vaughan, David Tweedie, Jin Jung, Hee Kyung Kim, Ho-Il Choi, Dong Seok Kim, Julie A. Mattison, Nigel H. Greig

**Affiliations:** 1 Translational Gerontology Branch, Intramural Research Program, National Institute on Aging, National Institutes of Health, Baltimore, MD USA; 2 SoBran BioSciences, SoBran Inc., Burtonsville, MD USA; 3 Translational Gerontology Branch, National Institute on Aging, National Institutes of Health, Dickerson, MD USA; 4 Peptron Inc., Yuseong-gu, Daejeon, Republic of Korea

**Keywords:** Metabolism, Neurodegenerative diseases

## Abstract

The time-dependent (30 min - day 84) plasma profile of PT320, a sustained-release (SR)-Exenatide formulation under clinical development for treatment of neurodegenerative disorders, was evaluated in nonhuman primates after a single subcutaneous dose and was compared to Bydureon. Exenatide release from PT320 exhibited a triphasic pharmacokinetic profile. An initial peak occurred at 3 hr post-administration, a secondary peak at 5 days, and achievement of Exenatide steady-state plasma levels from day 10–28. Systemic exposure increased across PT320 doses, and Exenatide levels were maintained above the therapeutic threshold prior to achieving a steady-state. In contrast, Exenatide release from Bydureon exhibited a biphasic profile, with an initial plasma peak at 3 hr, followed by a rapid decline to a sub-therapeutic concentration, and a gradual elevation to provide a steady-state from day 35–49. Exenatide total exposure, evaluated from the area under the time-dependent Exenatide concentration curve, was similar for equivalent doses of PT320 and Bydureon. The former, however, reached and maintained steady-state plasma Exenatide levels more rapidly, without dipping to a sub-therapeutic concentration. Both SR-Exenatide formulations proved well-tolerated and, following a well-regulated initial release burst, generated steady-state plasma levels of Exenatide, but with PT320 producing continuous therapeutic Exenatide levels and more rapidly reaching a steady-state.

## Introduction

The incretin glucagon-like peptide 1 (GLP-1) is a gastrointestinal peptide hormone and proteolytic product of proglucagon. It is predominantly generated within and released by intestinal enteroendocrine L cells to potentiate glucose-dependent insulin secretion by pancreatic β-cells following food ingestion^1,2^. Further to its well-characterized insulinotropic function and other actions on glucose homeostasis, produced by suppressing glucagon secretion from pancreatic *α*-cells, slowing gastric emptying and inducing satiety to reduce food intake and body weight, GLP-1 confers glucose sensitivity to glucose-resistant β-cells and provides trophic support by stimulating pancreatic β-cell proliferation and neogenesis, and by reducing β-cell apoptosis^3,4^. As the glucose homeostasis actions and enhancement of insulin signaling provided by GLP-1 are largely preserved in type 2 diabetes mellitus (T2DM), agents that activate the GLP-1 receptor (GLP-1R), a class B G protein–coupled receptor (GPCR) that mediates the action of GLP-1, have been developed for the treatment of T2DM; they appear to be well-tolerated, efficacious and widely used^5–9^. Furthermore, as the GLP-1R has been found expressed across a wide variety of other organs, such as on neurons within the brain and peripheral nervous system and within all four chambers of the heart, GLP-1R agonists are currently being evaluated across an increasing number of other diseases^10–15^.

GLP-1 has an extremely short half-life (T_1/2_ 1.5 min) within the circulation following either its physiological release or direct administration, with proteolysis, principally by DPP-4 and renal clearance having major roles. DPP-4 cleaves the amino-terminal dipeptide from GLP-1 to reduce its activity^1–9,16,17^. The identification of this led to the strategies for (i) modifying the peptide to lower proteolysis/renal clearance to generate longer-acting incretin mimetics and (ii) to elevate endogenous GLP-1 levels by using DPP-4 inhibitors. In relation to the former, an increasing number of GLP-1R agonists have been developed for clinical use and, as trials and experience with them grow, it will become clearer whether one has advantages over another within specific clinical settings. A practical way to categorize clinically available GLP-1R agonists is based on their duration of action. Centered on their pharmacokinetic/ pharmacodynamic profile, they can be divided into short-acting (Exenatide (*Byetta*): T_1/2_ 2.4 hr^18^, and lixisenatide T_1/2_ 3 hr^19^: administered twice and once daily, respectively) and long-acting GLP-1R agonists (Exenatide long-acting release (LAR (*Bydureon*)), liraglutide T_1/2_ 13 hr, albiglutide T_1/2_ 6–7 days, and dulaglutide T_1/2_ 4 days^20,21^: administered from once daily to weekly (Supplemental Table 1)). A key distinction between short- and long-acting GLP-1R agonists is that, when administered in line with their approved dosing schedule, the former group is subject to wide fluctuations in the plasma concentration of active compound, whereas the latter provides a more stable and sustained GLP-1R activation^20,21^.

**Table 1:** Pharmacokinetic parameters of Exenatide following a single subcutaneous administration of PT320 (0.4 and 1.1 mg/kg) and Bydureon (1.1 mg/kg) to nonhuman primates.

	PT320 0.44 mg/kg (A)	PT320 1.1 mg/kg (B)	Bydureon 1.1 mg/kg (C)
C_Ipeak_ (pg/ml)	924.5 ± 62.9 (*B*,*C*)	1740.2 ± 86.1 (*A*)	1960 ± 167.8 (*A*)
T_Ipeak_ (hr)	1.75 ± 0.7	3	3
C_Itrough_ (pg/ml)	72.1 ± 10.9 (*B*,*C*)	137.7 ± 21.7 (*A*,*C*)	8.6 ± 3.2 (*A*,*B*)
T_Itrough_ (hr)	60 ± 12	72	24
C_Max_ (pg/ml)	1227.9 ± 251.3	1535.7 ± 167.7	1617.4 ± 272.5
T_Max_ (hr)	438 ± 66 (18.25 days) (*C*)	416 ± 88 (17.3 days) (*C*)	966 ± 80.4 (40.25 days) (*A*,*B*)
Approx. steady-state	Day 10–28	Day 10–28	Day 35–49
T_Lag_ (hr)	436 ± 66 (18.2 days) (*C*)	413 ± 88 (17.2 days) (*C*)	963 ± 80 (40.1 days) (*A*,*B*)
C_Ave_ (pg/ml)	586.4 (Day 0–35)	676.9 (Day 0–49)	658.7 (Day 0–56)
AUC: 0–24 hr	12,540.2 ± 1,229.2 (*B*)	19,990.0 ± 839.1 (*A*)	18,375.1 ± 2,712.6
AUC: 0–2016 hr (i.e., day 0–84)	490,291.4 ± 113,343.2 (*C*)	738,234.0 ± 109,532.3	848,585.2 ± 91,142.9 (*A*)
AUC: 240–672 hr (i.e., day 10–28)	350,090.2 ± 81,038.5	451,360 ± 111,800.4 (*C*)	182,033.8 ± 28383.2 (*B*)
AUC: 840–1176 hr (i.e., day 35–49)	10,486 ± 3,949 (*C*)	71,914 ± 34,376 (*C*)	373,149 ± 44,892 (*A*,*B*)

*C*: Bydureon 1.1 mg/kg group is significantly different from the comparison group (p ≤ 0.05).

*B*: PT320 1.1 mg/kg group is significantly different from the comparison group (p ≤ 0.05).

*A*: PT320 0.44 mg/kg group is significantly different from the comparison group (p ≤ 0.05).

C_Ipeak_: Initial peak plasma concentration associated with the initial release burst of Exenatide. T_Ipeak_: Time of C_Ipeak_.

C_max_: Maximal plasma concentration of Exenatide.

T_Max_: Time of C_Max_.

T_Lag_: T_Max_ - T_Ipeak_

C_Ave_: Average plasma concentration of Exenatide (calculated from time zero up to the time when plasma levels were greater than 50 pg/ml (considered the minimal therapeutic concentration^28^).

AUC: are under the Exenatide plasma concentration curve between the defined times.

Exenatide was the first approved GLP-1R agonist for T2DM as a twice daily medication (*Byetta*: 2005) and its extended release formulation, Exenatide as *Bydureon*, was approved in 2012 for once weekly dosing. Both are well-tolerated and remain widely used^5–9,21^. In contrast to liraglutide that reversibly binds to serum albumin, albiglutide that is the fusion product of two modified GLP-1 molecules with albumin, and dulaglutide that comprises two modified GLP-1 molecules joined by a linker to a modified human immunoglobulin heavy chain – each tailored to protect against DPP-4 activity and renal clearance, Exenatide as *Bydureon* is the same 39 amino acid peptide as used in *Byetta* but is encapsulated in poly(lactic-co-glycolic acid) (PLGA) microspheres to enable its sustained release from the subcutaneous reservoir generated at the injection site^21–24^. Modifications in how Exenatide is formulated into PLGA can transform the release kinetics and the resulting pharmacokinetics of the drug^23–26^. In the current study, we evaluated a new sustained release (SR)-Exenatide formulation, PT320, with a longer (2 week) duration dosing profile that is under clinical development by Peptron Inc. (Daejeon, Republic of Korea) for the treatment of neurodegenerative disorders^27–29^. PT320 has similar properties to PT302, formerly developed by Peptron for the treatment of T2DM^30^, and is manufactured utilizing an ultrasonic spray drying process, termed SmartDepot™^31^, to generate microspheres that contain 2% Exenatide. These microspheres likewise comprise the polymer, PLGA. Notably, they are of small uniform size (20 μm diameter). This allows subcutaneous administration using a smaller needle size (27 to 30 gauge^31^, compared to 23 to 25 gauge for Bydurion) and, importantly, supports the rapid achievement of steady-state Exenatide levels. Additionally, their coating with L-lysine provides control of the initial burst of Exenatide released following PT320 subcutaneous administration. In the present study, time-dependent levels of Exenatide were evaluated in nonhuman primates following the subcutaneous administration of matched Exenatide doses within PT320 and Bydureon in order to compare their pharmacokinetic profiles.

## Results

**Figure 1:**
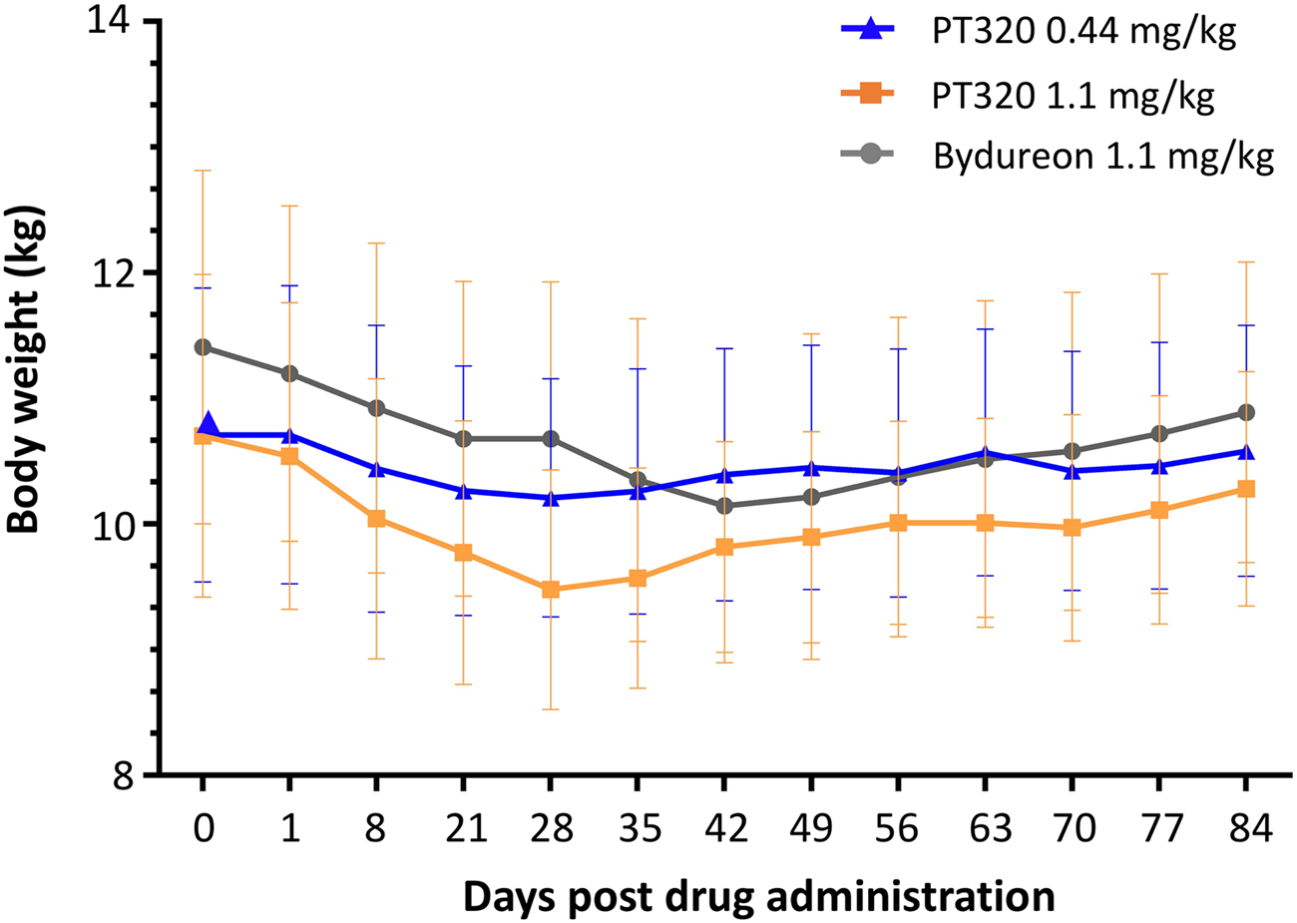
Time-dependent mean weight (kg) of rhesus monkey groups following Exenatide administration as either PT320 (0.44 or 1.1 mg/kg) or Bydureon (1.1 mg/kg), as compared to base line (day 0) weight prior to dosing.

Exenatide administration in the form of both PT320 (0.44 and 1.1 mg/kg) and Bydureon (1.1 mg/kg) proved to be well-tolerated at the doses evaluated, albeit hard nodules (in general 2 × 2 cm in size) were evident within the subcutaneous injection site across groups (3 of 4: PT320 0.44 mg/kg; 4 of 4: PT320 1.1 mg/kg; 4 of 4: Bydureon 1.1 mg/kg). For one animal in the PT320 1.1 mg/kg group (Monkey #12), the nodule broke the skin surface and resulted in drug loss (occurring at approximately weeks 3 and 4), and hence this animal was removed from the pharmacokinetic analysis. The mean body weight of rhesus monkeys is shown in Fig. 1, which decreased across all groups, reaching a trough between weeks 4 to 6 (day 28 to 42), and recovering nearly to baseline levels by week 12 (day 84).

**Figure 2:**
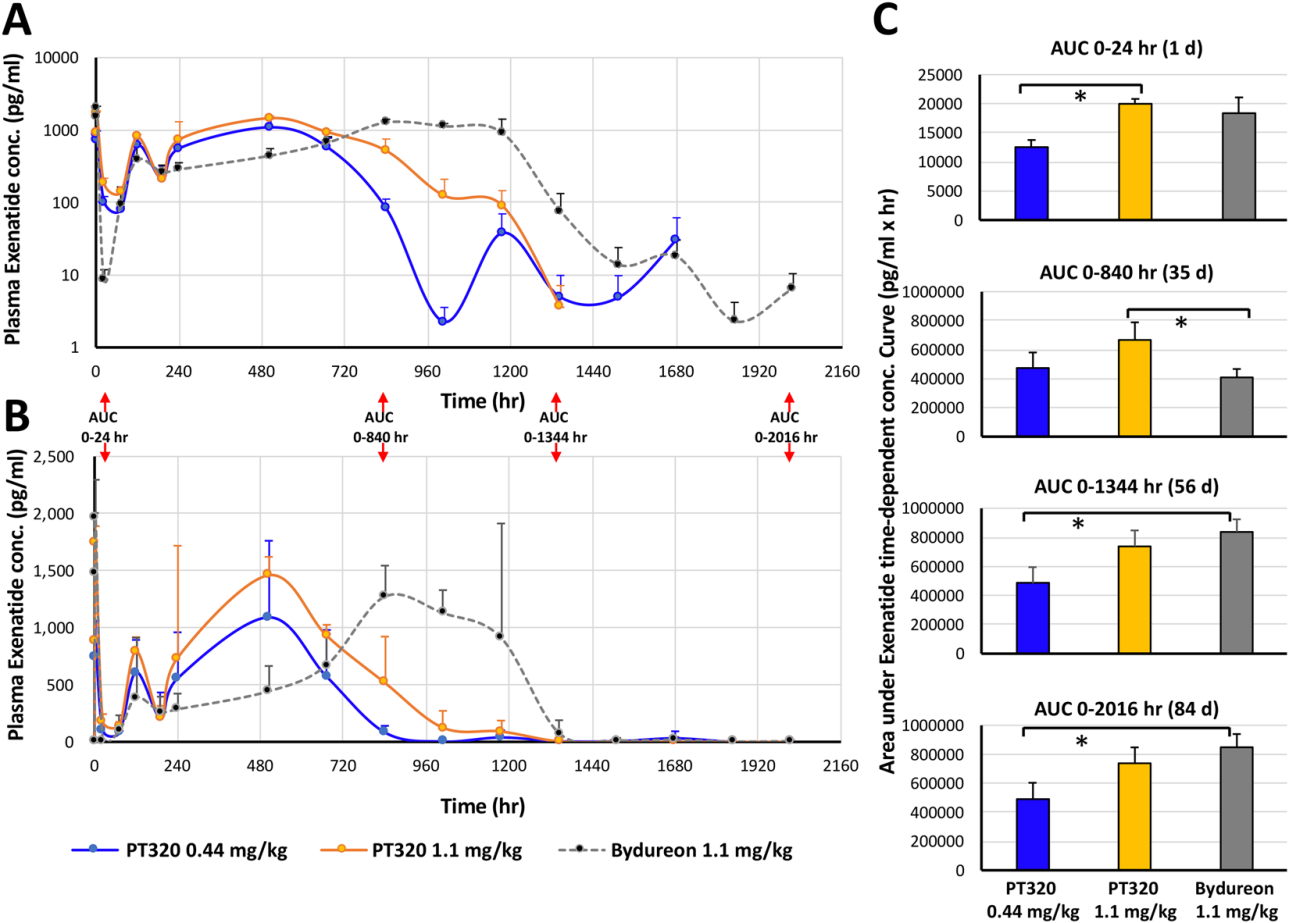
Time-dependent plasma concentrations of Exenatide following a single subcutaneous dose of PT320 (0.44 or 1.1 mg/kg) or Bydureon (1.1 mg/kg) to nonhuman primates. (**A**) Classical semi-log plot of plasma Exenatide levels by treatment group from 30 min to 84 days (0.5 to 2016 hr). (**B**) Linear. Plot of the same data to emphasize the different times that steady-state Exenatide levels were achieved following administration of either PT320 (blue and orange lines) vs. Bydureon (gray). (**C**) Plot of AUC values at the times noted in (**A** and **B**). Values are means ± SEM, *p < 0.05.

An initial release burst of Exenatide was evident in plasma as early as 30 min after administration of either dose of PT320 or of Bydureon. Illustrated in Fig. 2A is a semi-log plot of plasma Exenatide (pg/ml) levels versus time (hr) that combines animals within each of the three groups (PT320 0.44 and 1.1 mg/kg, and Bydureon 1.1 mg/kg). Following this initial Exenatide release, plasma levels declined across all treatment groups by 24 hr, reaching a trough (C_Itrough_) between 60 and 72 hr. For animals administered either dose of PT320, a secondary peak was consistently evident at 5 days, and approximately steady-state Exenatide plasma levels were then achieved from days 10 to 28 post administration (i.e., from approx. 1.5 to 4 weeks). In contrast, following Bydureon administration, plasma levels of Exenatide gradually and time-dependently increased to achieve an approximate steady-state concentration between day 35 to 49 (5 to 7 weeks) post injection; rapidly declining at week 8 and being minimal thereafter.

**Figure 3:**
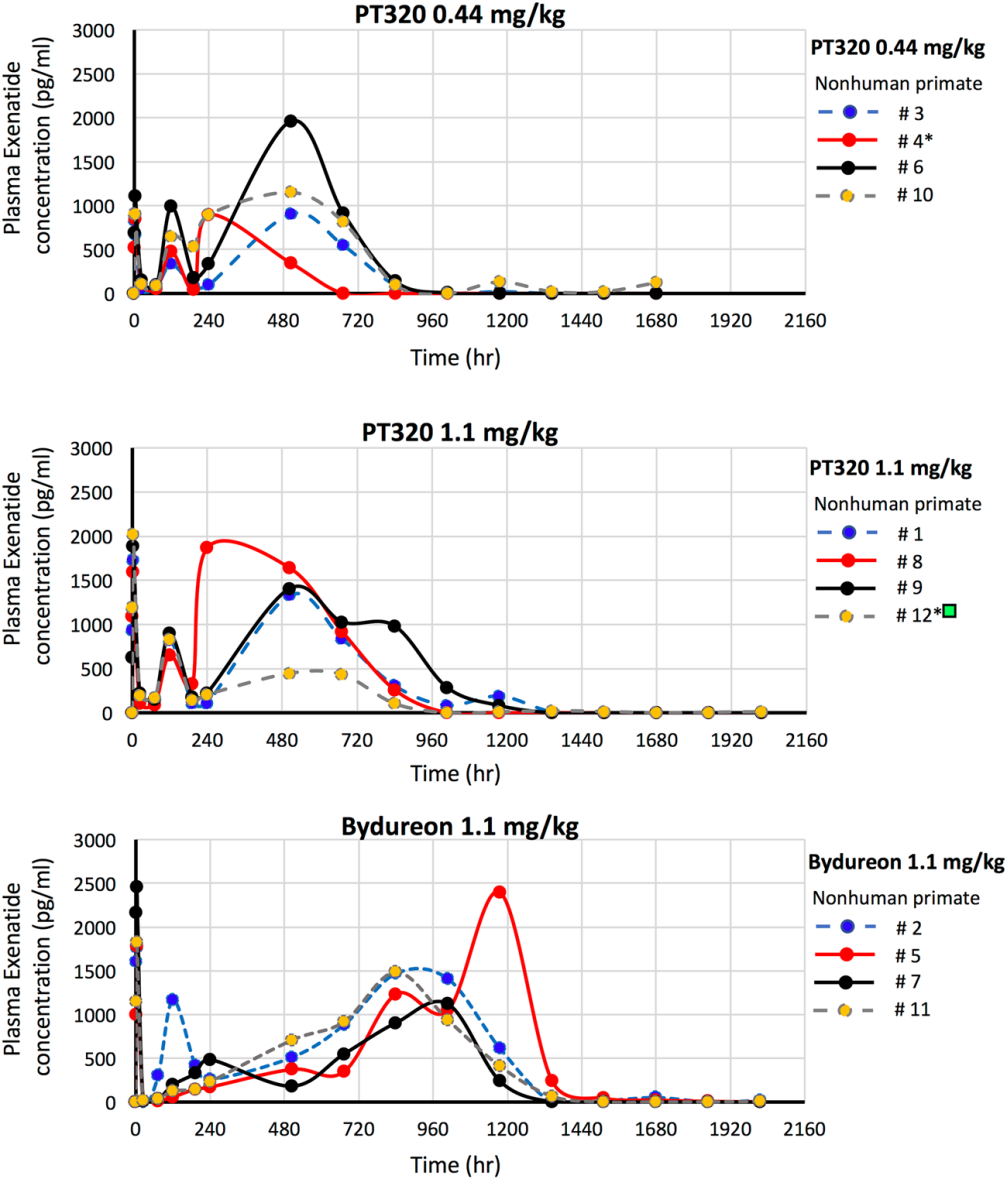
Linear time-dependent plasma Exenatide concentration plots for individual nonhuman primates administered a single subcutaneous dose of (**A**) PT320 (0.44 mg/kg), (**B**) PT320 (1.1 mg/kg) and (**C**) Bydureon (1.1 mg/kg). *Non human primates #4 and #12 demonstrated anti-Exenatide antibodies at 6 and 12 week evaluation times.  Nonhuman primate #12 was excluded from analysis of pharmacokinetic parameters as drug loss occurred at the skin surface at approximately week 3 to 4 (504 to 672 hr).

Figure 3 demonstrates linear time-dependent curves of Exenatide levels in plasma for each nonhuman primate, which allows an appreciation of the variance between individual animals administered either dose of PT320 or Bydureon. Such variance is not uncommon in nonhuman primate as, phylogenetically close to humans, they possess substantial outbred genetic variability, as compared to rodents^32,33^. Notably, and shown in Table 1, is that the plasma Exenatide concentration and time associated with the initial release burst, C_ipeak_ and T_ipeak_, following subcutaneous administration is remarkably similar between the matched 1.1 mg/kg doses of PT320 (1740.2 ± 86.1 pg/ml at 3 hr) and Bydureon (1960 ± 167.8 pg/ml at 3 hr) (p = 0.35). Likewise, the maximal concentrations, C_max_, achieved by these different formulations are also comparable; 1535.6 ± 167.7 and 1617.4 ± 272.5 pg/ml (p = 0.83), respectively. However, the time to achieve these, T_max_, was significantly shorter for PT320 compared to Bydureon (416 ± 88 h (i.e., 17.3 days) vs. 966 ± 80.4 h (i.e., 40.25 days), p = 0.006), and is reflected in the different time-dependent AUC values (Table 1 and Fig. 2B).

A notable difference between Exenatide administered as PT320 or as Bydureon is the depth of the decline in plasma levels of Exenatide that followed the initial release burst. Whereas the time-dependence of the decline was similar across formulations, reaching a trough between 24 and 72 hr (T_Itrough_), the lowermost Exenatide plasma concentration (C_Itrough_) maintained by Bydureon was significantly less (8.6 ± 3.2 pg/ml at 24 hr) than that achieved by PT320 (0.44 mg dose: 72.1 ± 10.9 pg/ml at 60 hr; 1.1 mg dose: 137.7 ± 21.7 pg/ml at 72 hr) (Table 1).

Also, of note is the similarity in the shape of the time-dependent Exenatide concentration curves generated by the two evaluated doses of PT320 (0.44 and 1.1 mg/kg) in nonhuman primates (Figs. 2 and 3). Whereas the times associated with the Exenatide initial release burst, subsequent trough and maximal concentration, T_ipeak_, T_Itrough_ and T_max_, were similar for the PT320 0.44 and 1.1 mg/kg doses (specifically, T_ipeak_: 1.75 ± 0.7 vs. 3.0 ± 0.0 hr (p = 0.2), T_Itrough_: 60 ± 12 hr vs. 72 ± 0.0 hr (p = 0.4); T_max_: 438 ± 66 hr (18.25 days) vs. 416 ± 88 hr (17.3 days) (p = 0.85), respectively), the concentrations associated with these demonstrated dose-dependence (C_ipeak_: 924.5 ± 62.9 vs. 1740.2 ± 86.1 pg/ml (p = 0.0005); C_Itrough:_ 72.1 ± 10.9 vs. 137.7 ± 21.7 pg/ml (p = 0.03); C_max_: 1227.7 ± 251.3 vs. 1535.6 ± 167.7 pg/ml (p = 0.39), respectively).

As the development of anti-Exenatide antibodies is not uncommon in human clinical studies^34^ due to a low homology of Exenatide to native GLP-1, we evaluated the development of such antibodies by Sandwich ELISA across all animals from plasma samples obtained at 6 and 12 weeks post PT320 or Bydurion administration. As illustrated in Table 2, two of 12 animals developed a positive titer that was present at both times (specifically, nonhuman primates #4 and #12).

**Table 2:** Anti-Exenatide antibodies evaluated in plasma at 6 and 12 weeks post subcutaneous Exenatide administration in the form of either PT320 (0.44 or 1.1 mg/kg) or Bydureon (1.1 mg/kg) to nonhuman primates. (+): positive antibody titer; (−): no antibody titer.

Nonhuman primate	Anti-Exenatide antibody (1:25)	
	6 weeks	12 weeks
# 1	(−)	(−)
# 2	(−)	(−)
# 3	(−)	(−)
# 4	(+)	(+)
# 5	(−)	(−)
# 6	(−)	(−)
# 7	(−)	(−)
# 8	(−)	(−)
# 9	(−)	(−)
# 10	(−)	(−)
# 11	(−)	(−)
# 12	(+)	(+)

## Discussion

The goal of the current study was to evaluate the pharmacokinetic properties of a single-dose of PT320 in healthy nonhuman primates. PT320 is a form of PT302^30^ that is being developed for neurological disorders^27^. Specifically, we characterized the time-dependent levels of plasma Exenatide following two single doses of PT320 (1.1 and 0.44 mg/kg) to provide an initial evaluation of dose-dependence. Additionally, we compared the higher PT320 dose to a matched single dose of Bydureon (1.1 mg/kg) to evaluate the equivalences of these two different SR Exenatide formulations. Systemic Exenatide exposure was measured periodically for 84 days (2016 hr) following a single subcutaneous dose of these formulations. These doses were based on a prior Bydureon study in nonhuman primates (Cynomolgus monkeys) in which single doses of 0.11, 0.44 and 1.1 mg/kg were evaluated from 0.5 hr to 71 days, and proved to be well-tolerated^35^. The minimally effective concentration of Exenatide in humans has been reported as approximately 50 pg/ml, as this is the level required to reduce fasting plasma glucose levels^36^. In using 50 pg/ml as a ‘cutoff limit’, the PT320 0.44 mg/kg administration provided a therapeutic level of dosing for up to 31.5 days (756 ± 84 hr), the PT320 1.1 mg/kg dose for up to 44 days (1064 ± 112 hr), and the Bydureon 1.1 mg/kg dose for 52 days (1260 ± 48.5 hr) in nonhuman primates (with no significant difference in this duration between the 1.1 mg PT320 and Bydureon formulations; p = 0.13). These results are in accord with data from human studies evaluating the pharmacokinetics of Exenatide release from PT302 in which concentration-dependent release was measured for up to 55 days following a single subcutaneous dose of PT302^30^, and for some 60 days (8.5 weeks) following Bydureon^8,37^ and, additionally, are consistent with weekly to biweekly dosing^38^.

In the clinical setting for the treatment of T2DM^1,5–8^ as well as for other disorders^10–14^, Exenatide is routinely administered chronically; over months to years duration. This would involve once weekly dosing of Bydureon^5–9^ and either once weekly or once every other week dosing for PT320^27,30^. Albeit not the focus of our single dose study, such multiple dosing would be expected to impact plasma Exenatide levels achieved at 7 and 14 days onwards, after Bydureon and PT320, respectively, and to maintain steady-state levels over a far longer duration. Importantly, Exenatide plasma levels associated with multiple dosing of either Bydureon or PT320 would be similar to that achieved with a single dose prior to 7 and 14 days, respectively, as only a single dose would have been administered at these times.

A consistent initial peak in plasma Exenatide was noted following PT320 administration to nonhuman primates within 1.75 to 3 hr, which is comparable to the characteristic peak seen in humans that occurs within 4 hr of dosing^30^. This first phase is caused by the ‘burst release’ of Exenatide occurring from the PLGA nanospheres in which the drug is encapsulated. This predominantly derives from Exenatide on or close to the surface that enters the systemic circulation on initial nanosphere hydration prior to the onset of polymer erosion-associated Exenatide release^25,39^. An initial peak in plasma Exenatide was likewise evident following Bydureon administration to nonhuman primates at 3 hr, in line with human studies in which a peak at 2.1 to 5.1 hr was noted^37^. Our study indicates that the concentration of the initial burst release in nonhuman primates appeared to be well-regulated across both Exenatide SR formulations, and was dose-dependent, with the C_Ipeak_ value approaching that of the C_max_ value (PT320 0.44 and 1.1 mg/kg 75% and 113%, and Bydureon 1.1 mg/kg 121%); and the variance around the C_Ipeak_ value is relatively small (6.8%, 4.9% and 8.6%, respectively re: SEM as a percent of the mean value). This initial burst peak was relatively short in duration, with Exenatide plasma concentrations declining rapidly by 24 hr to provide a time-dependent concentration of less than 3% of the total exposure to Exenatide (AUC_0-24_ hr ÷ AUC_0-2016_ hr). This is in accord with studies of PT320 in humans in which the value of C_Ipeak_ was likewise comparable to C_Max_, and the initial Exenatide burst represented only a small fraction of the total drug exposure (1.2% to 3.8% over the initial 12 hr^30^. Controlling the initial release of a drug from a SR preparation is a key parameter in the development of an extended release drug delivery system to rapidly achieve a therapeutic concentration without inducing an unregulated spike in drug level to potentially provoke an adverse action^24,25,39^, such as nausea or vomiting, which are not uncommon following the initial use of immediate release Exenatide (*Byetta*)^6,8,9,21,22^.

Also evident from our time-dependent analysis of plasma Exenatide concentrations generated by PT320 or Bydureon in nonhuman primates (Fig. 2A) is the short ‘lag phase’ (i.e., the decline in drug levels released into plasma) following the initial burst peak prior to the generation of relatively stable Exenatide levels. A key difference between the matched 1.1 mg PT320 and Bydureon doses was the depth of the decline in plasma Exenatide; falling to a sub-therapeutic concentration for Bydrueon (C_Itrough:_ 8.6 ± 3.2 pg/ml) at 24 hr across all (4/4) nonhuman primates and persisting at a sub-therapeutic level in 3/4 animals at 72 hr (Fig. 3C). In contrast, plasma Exenatide levels declined to a C_Itrough_ of 137.7 ± 21.7 pg/ml in 1.1 mg PT320 animals, with none from either PT320 doses declining below a therapeutic level (50 pg/ml^36^).

A secondary peak in plasma Exenatide levels was evident at 5 days following either dose of PT320 and, after a further decline of short duration, Exenatide levels reached and maintained a relatively steady-state concentration from days 10 to 28. The difference between the C_Ave_ and C_Max_ values was in the order of 2-fold (from Table 1; PT320 0.44, PT320 1.1 and Bydureon 1.1 mg/kg: 2.1-, 2.3- and 2.5-fold, respectively). A smaller disparity between C_Ave_ and C_Max_ for a drug delivery system is associated with superior extended release while concurrently maintaining the peptide in a stable form *in vivo*^23,24^.

A further major difference between Exenatide released from PT320 and Bydureon administration was related to the time required to reach steady-state plasma levels in nonhuman primates. For both PT320 doses this was achieved within 10 days and lasted up to day 28 (18 days duration), with plasma Exenatide levels never falling below the minimal therapeutic concentration of 50 pg/ml^36^ prior to achieving the steady-state. In contrast, Bydureon achieved steady-state levels of plasma Exenatide from approximately day 35 to 49 (14 days duration). Bydureon briefly declined to sub-therapeutic levels following the initial burst phase, and then gradually increased over time (Fig. 3C). Notable is the equivalence of the concentrations of Exenatide achieved for the matched 1.1 mg/kg doses of PT320 and Bydureon, whether the C_Ipeak_, C_Max_ or C_Ave_ values, which were remarkably similar (Table 1). As noted, the time (T_Max_) to achieve the C_Max_ was shorter for PT320, which is reflected in the AUC values (Table 1 and Fig. 2B). Also evident is that the C_Ipeak_, C_Itrough_, C_Max_ and C_Ave_ values demonstrate dose-dependence for both the PT320 0.44 and 1.1 mg/kg administrations, with the maintenance of the times to achieve these (Table 1). In comparison, in human subjects with T2DM administered PT320 the Exenatide C_Max_ occurred between 23 and 27 days, and demonstrated dose-dependence^30^. For Bydureon in humans with T2DM, the C_Max_ occurred between 39 to 48 days following administration and, likewise, was dose dependent^37^.

Following the initial burst release phase of Exenatide from the PLGA microsphere surface of either PT320 or Bydureon, the microspheres hydrate, adhere to one another, and create an *in situ* matrix drug reservoir. Continuous diffusion from this matrix results in the secondary release phase of Exenatide into plasma that, followed by the hydrolysis and erosion of the PLGA polymer, results in the steady-state Exenatide third release stage^23–25,39^. The PLGA polymer hydrolysis products, lactic and glycolic acids, are ultimately eliminated as carbon dioxide and water^23^. Whereas PLGA is a biodegradable and biocompatible medical polymer with a well-established safety profile as an excipient in a wide variety of controlled release drugs^25,39,40^, the appearance of subcutaneous injection site reactions for Bydureon is reported as 17.1% from clinical data presented within the US approved prescribing information^41^. When present, these reactions may occur after the first dose (70%), and cause local skin discoloration (70%), pain or discomfort (56%), pruritus (48%), warmth (33%), and swelling (30%) at the injection site. Most cases reported nodules characterized as hard, subcutaneous, lumps, masses, or indurations^41^, with a minority (22%) of cases reporting a concurrent abscess. The occurrence of injection site induration and pruritus was also noted in T2DM subjects administered PT320, and is described to be dose-dependent and to naturally resolve^30^. In our nonhuman primate study, such Bydureon and PT320 associated nodules were evident across all animals (12 of 12) with one demonstrating an abscess (1 of 12), and all self-resolved. In contrast, Bydureon in humans is routinely administered as a dose of 2 mg in a 0.65 ml injection volume (or 0.85 ml for Bydureon BCISE)^22^, and hence the doses and corresponding injection volumes are substantially larger in our nonhuman primate study (mean values: 1.7 and 4.1 ml for PT320 0.4 and 1.1 mg/kg doses, and 3.5 ml for Bydureon 1.1 mg/kg dose), and likely resulted in a higher injection reaction site incidence and unusual loss of drug in nonhuman primate #12.

Our study was specifically designed to follow time-dependent Exenatide levels achieved by the sustained release formulations PT320 and Bydureon, rather than to evaluate the Exenatide elimination half-life from plasma – which is generally unchanged when the same drug entity is administered either by immediate or sustained release preparation^42,43^. Notable across both PT320 doses and Bydureon administration in our nonhuman primate study is the rapid decline in plasma Exenatide levels at the end of steady-state, coinciding with the exhaustion of the drug reservoir. This is in accord with a prior pharmacokinetic study of subcutaneous immediate release Exenatide in rhesus monkeys in which the terminal disappearance half-life from plasma ranged from 0.78 to 1.94 hr.^44^. Studies across animal species and humans indicate that Exenatide, whether administered by immediate or sustained release, is predominately eliminated by the kidney via glomerular filtration with subsequent enzymatic degradation^45^. Studies in nephrectomized rats demonstrated that Exenatide slowly disappeared from plasma signifying the occurrence of a non-renal clearance component^46^. This may involve target-mediated drug disposition^47^, encompassing the binding of Exenatide to the GLP-1R, and the formation of Exenatide-GLP-1R complexes that either dissociate or become internalized and targeted for receptor-mediated endocytosis and degradation^48^.

In the light of reports of anti-Exenatide antibody development in human studies focused on Exenatide use in T2DM^34,37^ as well as in preclinical studies^31^, we evaluated whether such an occurrence transpired in our nonhuman primate study by evaluating plasma samples at 6 and 12 weeks. Two of 12 animals (16.7%) demonstrated a positive antibody titer. Interestingly, these two (nonhuman primates #4 and #12 in the PT320 0.44 and 1.1 mg/kg groups, respectively) were associated with lower values of plasma Exenatide (Fig. 3). Bydureon was reported to induce anti-exenatide antibodies as early as day 29 at ≥0.44 mg/kg dose in nonhuman primate studies undertaken by the original manufacturer [35 (page 57)], in a 3 month study involving once weekly administration. Low anti-Exenatide antibody titers (≤125) have been reported in 32% and 45% of T2DM patients administered Exenatide either as BID Byetta or as once weekly Bydureon, respectively, within 24 to 30 weeks of treatment, and are considered to not impact efficacy^34,37^. Higher-titer antibodies (≥625) less commonly occur (5% and 12% of patients, respectively) and may diminish Exenatide efficacy^37^. Such antibodies do not appear to cross-react with human GLP-1 or glucagon, or influence the safety profile of Exenatide formulations^37^.

In summary, our evaluation of a matched dose of the SR-Exenatide formulations PT320 and Bydureon (1.1 mg/kg) in nonhuman primates demonstrated a similar, well-regulated, initial burst release of Exenatide into plasma within 3 hr. This was followed by a lag phase and a secondary and tertiary release stage of Exenatide to provide reasonably steady-state levels in plasma. Whereas the plasma levels of Exenatide associated with C_Ipeak_, C_Max_ and C_Ave_ were equivalent, importantly the time to achieve C_Max_ (T_Max_) and the steady-state occurred significantly earlier for PT320. Furthermore, the dramatic decline in plasma Exenatide levels that followed the C_Ipeak_ and resulted in the C_Itrough_ was attenuated in the PT320 formulation and, unlike Bydureon, continuously maintained therapeutic drug levels prior to reaching steady-state delivery. Evaluation of a lower PT320 dose (0.44 mg/kg) in nonhuman primates demonstrated dose-dependence in relation to C_Ipeak_, C_Itrough_, C_Max_ and C_Ave_ values, with maintenance of the times to achieve these and the duration of the steady-state. Notably, our C_Max_ and AUC (0–2160 hr) values for Bydureon 1.1 mg/kg are in accord with prior preclinical nonhuman primate data that supported the agent’s approval for clinical use (see: C_Max_ and AUC (0–1704 hr) in^35^). For treatment of disorders involving the use of an SR-Exenatide formulation in which the rapid generation and long-term maintenance of therapeutic plasma and target levels is desired, as could be considered in the experimental treatment of mild and moderate head injury^29,49–53^ or acute ischemic injury^54–57^ (see for example clinical trials: NCT03287076 and NCT02829502), the pharmacokinetic characteristics of PT320 may prove particularly important; associated with the rapid achievement and maintenance of therapeutic Exenatide levels in plasma. For chronic disorders, such as T2DM and PD in which Exenatide has demonstrated value^1–3,5–11,13,14,20–22,58^, both sustained release formulations would appear to be valuable to achieve and maintain long-term steady-state drug levels through the dosing of Bydureon once weekly and PT320 either once every two weeks or once weekly.

## Materials and Methods

### Study subjects

Nonhuman primates were used in this study to provide a closer translational link in relation to evaluating a new drug formulation destined for use in humans, as compared to studies in rats^33,35^. Animals included 12 male (4–11 years old) rhesus macaques (*Macaca mulatta*) randomized into three treatment groups (n = 4). Monkeys were maintained at the National Institutes of Health Animal Center (Poolesville, MD) and housed in standard primate caging with a controlled temperature and humidity and a 12-hr light cycle. Commercially prepared monkey chow was distributed twice per day along with daily food enrichment, and water was available *ad libitum*. Monkeys were observed daily for food consumption and overall well-being. Animal husbandry and all experimental procedures complied with the National Institutes of Health Guide for the Care and Use of Laboratory Animals and were conducted under approved protocols by the National Institute on Aging Intramural Research Program Animal Care and Use Committee.

### Experimental design

At each procedure, monkeys were restrained with ketamine (3–5 mg/kg, IM), which was supplemented with partial doses of ketamine, as needed, to maintain anesthesia. Following an initial baseline blood draw to obtain background signal levels for Exenatide ELISA evaluations, one of three pharmaceutical grade treatments was administered subcutaneously into the abdomen (with the selected doses representing the active pharmaceutical ingredient (API) within each Exentatide sustained release formulation – to ensure matching of the Exenatide in PT320 and Bydureon (this was undertaken to account for the different percents of API, approx. 2% and 5%, respectively, within these formulations). The treatment groups included (i) PT320 0.44 mg/kg (mean body weight 10.45 ± 1.18 kg; injection volume 160 ul/kg), (ii) PT320 1.1 mg/kg (mean body weight 10.32 ± 1.15 kg; injection volume 400 ul/kg), and (iii) Bydureon 1.1 mg/kg (mean body weight 11.02 ± 1.45 kg; injection volume 320 ul/kg). A 4.0 ml blood sample was collected into an EDTA vacuum tube at 0.5, 3, and 24 hr, at days 3, 5, 7, and 10, and then once per week throughout the 12-week study (a total of 18 time points per animal). Blood samples were immediately placed on wet ice, centrifuged within 30 min (10,000 G, 2 min, 4 °C) and, following plasma removal, separate aliquots were frozen and stored at −80 °C.

### Exenatide assay

Exenatide levels were quantified by using the Peptron Exendin-4 EIA Kit (Peptron Inc., Daejeon, South Korea). Each sample was evaluated in duplicate at a volume of 50 ul each, with a plasma dilution of 1:10. Concentrations of Exenatide were subsequently determined from standard curves of newly prepared Exenatide, following preliminary studies to ensure that all results fell within the linear range of the plasma standard curves. Notably, this Exenatide assay appears to lack cross-reactivity with glucagon, oxyntomodulin, GLP-1 or GLP-2.

### Anti-Exenatide antibody measurement

As both PT320 and Bydureon are slow-release formulations of Exenatide and our study extended over a period of 12 weeks, we evaluated plasma anti-Exenatide antibody levels at both week 6 and week 12 using a previously developed homemade Sandwich ELISA^28^. In brief, ELISA plates were coated with Exendin-4 (concentration: 2 ug/ml in coating buffer) at 4 °C overnight, and following blocking and washing steps, standards (mouse monoclonal anti-Exenatide antibody) and unknown samples with serial dilutions were added to the plate and incubated at room temperature for 1 h. After washing, biotinylated-Exenatide (concentration: 2 ug/ml) was added, and was followed by washing and SA-HRP detection. The titers of the anti-Exenatide antibody within the samples were then estimated by serial dilution of the plasma (to a maximum dilution of 1:225). We used results from the 1:25 dilution of all samples to determine whether a positive or negative development of anti-Exenatide antibodies had occurred across all 12 nonhuman primates evaluated in our study.

### Statistics and pharmacokinetic evaluation

The pharmacokinetic characteristics of PT320 and Bydureon were assessed by following Exenatide time-concentration profiles and pharmacokinetic parameters, which were analyzed with noncompartmental methods using WinNonlin (version 6.0; Pharsight Corporation, Cary, NC). The baseline (zero time) sample was quantified for each animal as a level of ‘background signal’, and this value was then subtracted from time-dependent values in the same animal. The pharmacokinetic parameters evaluated included (i) the initial peak concentration (C_Ipeak_) associated with the initial release burst of Exenatide, and the time to reach C_Ipeak_ (T_Ipeak_). (ii) The lowest concentration of Exenatide in plasma (C_Itrough_) achieved subsequent to the C_Ipeak_, and the time of this decline (T_Itrough_). (iii) The ultimate maximal Exenatide concentration (C_Max_), and time to reach it (T_Max_). (iv) The lag time between the achievement of C_Ipeak_ and C_Max_ (T_Lag_). (v) The area under the curve from 0 to the last time point (AUC_0–last_), as calculated by the linear trapezoidal and log-linear trapezoidal methods. The lower limit of quantitation (LLOQ) for Exenatide in our study was 3.1 pg/ml.

## Supplementary information

Characteristics of currently available US FDA approved GLP-1 receptor agonists

## Data Availability

The dataset analyzed during the current study are available from the corresponding author on reasonable request.
