# Supplementary material for: Pharmacokinetics of Exenatide in nonhuman primates following its administration in the form of sustained-release PT320 and Bydureon
Source: Sci Rep. 2019 Nov 20;9:17208. doi: 10.1038/s41598-019-53356-2 (PMC6868133; doi:10.1038/s41598-019-53356-2)
Supplement: Supplementary file 1 — Characteristics of currently available US FDA approved GLP-1 receptor agonists [file 41598_2019_53356_MOESM1_ESM.docx]

Supplementary Table 1

**Pharmacokinetics of Exenatide in nonhuman primates following its administration in the form of sustained-release PT320 and Bydureon**

Yazhou Li^1#^, Kelli L. Vaughan^2,3#^, David Tweedie^1^, Jin Jung^4^, Hee Kyung Kim^4^, Ho-Il Choi^4^, Dong Seok Kim^1,4^**^∆^**, Julie A. Mattison^2^**^∆^**, Nigel H. Greig^1^**^∆^***

^1^ Translational Gerontology Branch, Intramural Research Program, National Institute on Aging, National Institutes of Health, Baltimore, MD, USA.

^2^ SoBran BioSciences, SoBran Inc., Burtonsville, MD, USA.

^3^ Translational Gerontology Branch, National Institute on Aging, National Institutes of Health, Dickerson, MD, USA.

^4^ Peptron Inc., Yuseong-gu, Daejeon, Republic of Korea.

^#^ Equal contributions as first author

^∆^ Equal contributions as senior author

*Corresponding author: Nigel H. Greig, Email: [Greign@mail.nih.gov](mailto:Greign@mail.nih.gov)

Characteristics of currently available US FDA approved GLP-1 receptor agonists

| Common Name | Half Life (T_1/2_) | Typical Dose^a^ | Administration Schedule | FDA Approval |
| --- | --- | --- | --- | --- |
| **Short-Acting (< 24 hr)** [1,2] | | | | |
| Exenatide (as *Byetta*) | 2.4 hr | 5 – 10 μg | Twice Daily | 2005 |
| Lixisenatide | 3 hr | 10 – 20 μg | Daily | 2016 |
| **Long-Acting (> 24 hr)** [3-5] | | | | |
| Exenatide (as *Bydureon*) | 6 – 7 days | 2 mg | Weekly | 2012 |
| Liraglutide | 13 hr | 0.6 mg, 1.2 mg or 1.8 mg | Daily | 2010 |
| Albiglutide (discontinued) | 6 – 7 days | 30 or 50 mg | Weekly | 2014 |
| Dulaglutide | 4 days | 0.75 or 1.5 mg | Weekly | 2014 |
| Semaglutide | 6 – 7 days | 0.5 or 1 mg | Weekly | 2017 |

1. Oshima, I., Hirota, M., Ohboshi, C. & Shima, K. Comparison of half-disappearance times, distribution volumes and metabolic clear-ance rates of exogenous glucagon-like peptide 1 and glucagon in rats. *Regulatory Peptides* **21**, 85 –93 (1998).
2. Bray, G.M. Exenatide. *Am J Health Syst Pharm*.  **63**, 411–418 (2006).
3. Bain, S.C. The clinical development program of lixisenatide: a once-daily glucagon-like Peptide-1 receptor agonist. *Diabetes Ther*. **5**, 367-383 (2014).
4. Evans, M., Bain, S.C. & Vora, J. A systematic review of the safety of incretin-based therapies in type 2 diabetes. *Expert Rev Endocrinol Metab*. **11**, 217-232 (2016).
5. Glotfelty, E.J., et al. Incretin Mimetics as Rational Candidates for the Treatment of Traumatic Brain Injury. *ACS Pharmacol. Transl*. Sci. **2**, 66-91 (2019).
